# Impact of conditional cash transfer on households food security in Akwa Ibom State, Nigeria

**DOI:** 10.1371/journal.pone.0325594

**Published:** 2025-06-18

**Authors:** Otu W. Ibok, Iniobong E. Okon, Oscar P. Akpan, Imefon F. Isip

**Affiliations:** 1 Global Academy of Agriculture and Food Systems, University of Edinburgh, Edinburgh, United Kingdom; 2 Department of Agricultural Economics & Extension, Akwa Ibom State University, Obio-Akpa Campus, Uyo, Nigeria; 3 National Social Safety-Nets Coordinating Office (NASSCO), Uyo, Akwa Ibom, Nigeria; 4 Ministry of Agriculture & Food Sufficiency, Uyo, Akwa Ibom, Nigeria; Federal University of Agriculture Abeokuta, NIGERIA

## Abstract

The Nigerian government introduced the Household Upliftment Program (HUP) which is a conditional cash transfer scheme to help households improve consumption levels, reduce poverty and therefore prevent vulnerable households from becoming poorer. This paper investigated the impact of Condition Cash Transfer of HUP on household food security in Akwa Ibom State, Nigeria. The objectives were to assess the impact of CCT on protecting the beneficiary’s basic level of food consumption from becoming food insecure and also determine if CCT of HUP facilitate the beneficiary to invest in human and other productive assets. The paper utilized descriptive statistics, food security index, Likert scale and propensity score technique to analyse the research objectives. The findings showed that on average, benefitting and non-benefitting households spent about N31,917.78 ($76.46) and N34,898.67 ($83.60) respectively on food per month. Food insecurity was higher among benefiting households compared to non-benefiting households. The Average Treatment Effect on the Treated (ATET) estimator value was −0.2610. The negative value suggests that, on average, benefiting households spend 26.10% less on food consumption than non-benefitting households. The results imply that the current cash transfer program may not be effectively addressing food insecurity among beneficiaries. However, the CCT program facilitated the beneficiaries’ ability to invest in human and productive assets, enhanced their financial and social inclusion, opened doors to financial services previously inaccessible to many and fostered a sense of community among them. The paper concludes that while conditional cash transfer programs have the potential to positively impact beneficiaries, the government must reassess and potentially adjust the program to better address the current inflationary pressures and ensure its positive impact on the food security of the beneficiaries.

## I. Introduction

Food insecurity remains a critical global issue, prompting international bodies like the United Nations (UN) to address it through initiatives such as the Sustainable Development Goals (SDGs) launched in 2015, which aim to achieve zero hunger by 2030 [1]. Food security is crucial for the well-being and livelihood of citizens, as it ensures access to necessary nutrients, thereby contributing to social stability by reducing poverty, inequality, and conflict [2].

Nigeria, the most populous country in Africa and the sixth most populous in the world, faces a severe food insecurity crisis, exacerbated by rapid population growth. With Nigeria’s population surpassing 220 million, approximately 26.5 million people are projected to face acute hunger in 2024, a stark increase from the 18.6 million who were food insecure by the end of 2023 [3]. This was evident when the Nigerian President declared a state of emergency on food insecurity in July 2023 [3]. To combat this crisis, the government has implemented various policies and programs to enhance agriculture and food accessibility, including the National Social Investment Programme (NSIP) launched in 2015 [4].

Among NSIP’s initiatives is the Conditional Cash Transfer (CCT) scheme, also known as the Household Upliftment Program (HUP). This program aims to support poor and vulnerable households by providing NGN 5,000.00 monthly, contingent upon meeting specific conditions such as 80% school attendance for children, investment in farming activities, petty trading, and attendance of monthly training sessions organized by the national cash transfers office [4]. The overarching goal is to improve household consumption levels, foster savings habits, reduce poverty, and enhance the overall capacity of vulnerable households to become more self-sufficient [5].

The Household Uplifting Program (HUP) targeted the poorest households from 2016 to 2022, offering NGN 5000.00 monthly across the 36 states and the Federal Capital Territory [6]. As a social protection initiative, it aimed at achieving short-term poverty alleviation and long-term human capital development by encouraging investments in children’s health and education [7,8]. Similar programs in Latin America and Mexico have demonstrated significant success, prompting adoption in other countries to alleviate poverty, reduce household vulnerability to shocks, build human capital, and enhance nutritional and welfare outcomes [9].

HUP’s components in Nigeria include the Government Enterprise Empowerment Programme (GEEP), Home Grown School Feeding, N-power, and the National Cash Transfer Programme (CCT) [10]. Supported by the World Bank under the National Social Safety Nets Project (NASSP), HUP aims to provide financial assistance to poor and vulnerable Nigerian households as part of the country’s growth and social inclusion strategies [10,15]. HUP comprises three components: base Cash Transfer, Top-Up based on state-selected conditions, and Livelihood support [10]. The slogan “Beta Don Come,” meaning “better things have arrived,” encapsulates HUP-CCT’s objectives to improve household consumption, health, education, environmental sanitation, asset acquisition, and sustainable livelihoods [10].

The HUP, introduced by the Nigerian government, provides monthly cash transfers to poor and vulnerable households to improve their consumption levels and reduce poverty [4]. However, there is limited empirical evidence on the effectiveness of such programs in improving food security in Nigeria. Therefore, this study contributes to the broader academic discourse on food security, poverty alleviation, and social protection, adding to existing literature and fostering further investigations into effective interventions [11–14]. By assessing the relationship between cash transfers and food security, this research contributes to a deeper understanding of how financial support influences households’ access to food and their overall consumption status.

This study’s objectives examines whether CCTs under the HUP help protect beneficiary households from food insecurity and whether they facilitate investments in human and productive assets.

The paper is organized as follows: Section II reviews and discusses literature on the impact of conditional cash transfers, Section III presents the research methodology, Section IV describes the results and key findings, Section V discusses the implications of the findings, and Section VI concludes the study.

## II. Literature review

Conditional cash transfers (CCTs) have garnered significant attention as a social protection strategy aimed at combating poverty and enhancing various aspects of household well-being, including food security [7]. Extensive global evidence underscores the effectiveness of cash transfers in improving health and nutrition outcomes [15].

Studies examining the impact of CCTs on household food security highlight their potential benefits. [16] analyzed the effects of monthly cash grants targeted at ultra-poor households in Malawi, finding a five-percentage point increase in children’s school enrolment, higher educational expenditures, fewer absences, and a ten-percentage point decrease in external labour. Similarly, [17] conducted a comparative analysis of cash transfers, public works, and food transfers, noting that while comprehensive evidence comparing these mechanisms is limited, they observed improvements in households’ food consumption/expenditure by 13%, caloric acquisition by 8%, and asset holdings, including livestock and non-farm productive assets.

Further investigation by [18] into rural Niger revealed that households receiving food transfers experienced significant improvements in dietary diversity compared to those receiving cash transfers. However, cash transfer recipients were more inclined to make bulk purchases of grains and allocate more funds towards private transfers and debt repayment.

A systematic meta-review of CCTs’ impact on households with young children reported increased dietary diversity, decreased stunting, higher consumption of animal-sourced foods, and a reduced incidence of diarrhoea [19]. These findings suggest that CCTs can positively affect household nutritional status by improving food consumption and dietary diversity. Additionally, long-term benefits of CCTs include enhancements in educational outcomes, cognitive skill development, off-farm employment, and household incomes [20,21].

Despite these positive outcomes, the impact of CCTs on household food security is influenced by several factors, such as access to health services, clean water, hygiene practices, and household characteristics. For instance, [22] reviewed the long-term impact of CCT programs in Latin America, noting positive effects on household incomes and educational outcomes, but less pronounced impacts on food security. When combined with social health insurance schemes, CCTs and unconditional cash transfers were found acceptable to vulnerable families needing to mitigate tuberculosis treatment costs in Vietnam [23].

[24] assessed the impact of CCT programs, including the National Cash Transfer Programme (NCTP), across multiple countries such as Iran, Mexico, and Bangladesh. The analysis revealed that CCT programs reduced extreme poverty and improved nutritional, health, and educational outcomes. However, the replacement of food subsidies with cash transfers in Iran led to significant increases in household food insecurity. Similarly, UNICEF’s review of social cash transfer program evaluations in Africa demonstrated positive impacts on consumption, food security, and children’s nutritional outcomes, although inflation and price hikes could affect food purchasing power [25]. A rigorous literature review by ODI, covering 15 years (2000–2015) in low- and middle-income countries, further confirmed the positive impact of cash transfers on food security and nutrition, emphasizing the importance of program design and implementation features in determining their effectiveness [26].

In summary, while CCTs have shown considerable promise in enhancing household food security and overall well-being, their effectiveness is mediated by contextual factors and program-specific characteristics. The current study aims to build on existing literature by evaluating the impact of the Household Upliftment Program (HUP) in Akwa Ibom State, Nigeria, contributing to a deeper understanding of CCTs’ role in addressing food insecurity in diverse contexts.

## III. Methodology

This section describes the study area, sampling technique and size, method of data analysis, and ethical approval.

### Description of the study area

Akwa Ibom State is one of the 36 states in Nigeria. It is in the coastal southern part of the country, lying between latitudes 4°32′N and 5°33′N, and longitudes 7°25′E and 8°25′E. The state is in the South-South Geopolitical zone of Nigeria and is bordered on the east by Cross River State (nicknamed, The People’s Paradise), on the west by Rivers State (nicknamed, Treasure Base of the Nation) and Abia State (nicknamed, God’s own state) and on the south by the Atlantic Nation and the southernmost tip of Cross River State. Akwa Ibom boasts of a population of over five million people. The state was created in 1987 by Ibrahim Babangida, the then Head of State of the country, from the former Cross River State [27]. Akwa Ibom is currently the highest oil- and gas-producing state in the country (with an estimated production of 800,000 barrels per day). The state’s capital is Uyo, which has an estimated population of 500,000. Akwa Ibom remains a beautiful state that boasts of rich cultural values, aesthetic beauty and state-of-the-art facilities. Akwa Ibom is fast becoming a smart city that is keyed into building a more sustainable Nigeria with respect to the Sustainable Development Goals [28].

### Sampling technique and size

This study was conducted in 2022. A three-stage sampling procedure was used to select both benefiting and non-benefiting households for this study. Out of the thirty-one (31) Local Government Areas (LGA) in Akwa Ibom state, only nine (9) are benefitting from the Conditional Cash Transfer program, namely: Ukanafun, Onna, Ikono, Eastern Obolo, Nsit Atai, Nsit Ubium, Uruan, Orukanam, and Mkpat Enin.

In the first stage, all the names of these LGAs were included in a basket, and three LGAs were randomly drawn: Uruan, Mkpat Enin, and Nsit Atai. In the second stage, a list of the total number of beneficiaries of the cash transfer was obtained from the National Cash Transfer Office, Akwa Ibom State, Nigeria. Unfortunately, this data cannot be included here due to data access agreement restrictions. Recognizing that the study unit is households while the sampling frame is individuals, the research had to build a list of households that are beneficiaries of the conditional cash transfer program in the selected LGAs. The study worked with the local government officers in charge of Uruan, Mkpat Enin, and Nsit Atai to select households. During the regular fortnightly meetings between the beneficiaries and the local government officers, the researchers were able to meet and randomly select 100 households who volunteered for this study. From this list, 30 benefiting households were randomly selected from each of the three LGAs used in this study, totalling 90 benefiting households.

In the third stage, which involved selecting non-benefiting households, one community each for Uruan, Mkpat Enin, and Nsit Atai LGAs was randomly selected. In each of these communities, twenty non-benefiting households were selected, totalling 60 non-benefiting households for the study. In each household, either the household head or an adult was purposively selected for having adequate knowledge of the household consumption. Eventually, a total of 150 respondents were used for this study. A summary of the sampling size is presented in Table 1.

**Table 1:** Sampling size selection.

Selected LGA	Beneficiaries	Non-Beneficiaries
Uruan	30	20
Mkpat Enin	30	20
Nsit Atai	30	20
**Sub-Total**	**90**	**60**
**TOTAL**	**150**	

### Data collection tool

The primary data collection tool utilized for this research was questionnaires. All questions were administered in English. The questionnaire aimed to gather information on location, agricultural activities, household composition, food expenditure, sources of income, household assets, conditional cash transfers, and the benefits derived from the conditional cash transfer program.

### Method of data analysis

To assess the food security status of beneficiaries and non-beneficiaries in the study area, the study adopts the expenditure method used by [29]; [30]. Monthly food expenditure for beneficiaries was used to compute the per capita monthly expenditure. A food-secure household is one whose per capita food expenditure is above or equal to two-thirds of the mean per capita food expenditure of all households otherwise the household is food insecure. Equation 1 defines the food insecurity index used in this study to compute the food security status of both benefitting and non-benefitting households.


$$F_{i}=\frac{per\;capita\;food\;expenditure\;for\;the\;ith\;household}{\frac{2}{3}mean\;per\;capita\;food\;expenditure\;of\;all\;households}$$ (1)

Where: $F_{i}\;$ = food security index, when $F_{i}>1$ = food secure i^th^ household and $F_{i}<1$ = food insecure i^th^ household.

Using the food security index to compute the food security status, the household monthly food expenditure is divided by the household size of each household to derive the per capita food expenditure. After this, the total of the per capita food expenditure for all households (respondents) is divided by the total sample size to derive the mean per capita household expenditure. The food security threshold is computed by multiplying the mean per capita food expenditure by 2/3. Finally, the food security status of each household is derived by dividing their per capita food expenditure by the 2/3 mean per capita food expenditure. Hence, any household whose status is equal to or greater than one is regarded as food secure while household less than one is regarded as food insecure.

The impact derived from HUP was analyzed by asking the respondents to indicate whether or not they derived any or combination of the listed benefits such as payment of monthly upkeep allowance of five thousand naira, training and empowerment, new business establishment, business boost, lower level of dependency, increased psychosocial support, better inclusion in groups and increased self-confidence. Three-point Likert scale [31] was used to analyse this objective. Highly beneficial was scored as 3, beneficial 2, and not beneficial 1.

In analysing the effect of Conditional Cash Transfer (CCT) on household food security, propensity score matching technique was used. Propensity score for each household is defined as the conditional probability of being treated (i.e., being selected to receive the conditional cash transfer) given the vector of observed covariates [32–34]. Based on the assumption that observed covariates $X=(X_{1},\;X_{2},\;X_{3}\ldots ..X_{10}\;)$ and an indicator treatment group Z (Z = 1 if treated and Z = 0 if control) are assigned to each household. The propensity score (PS) for each household in this study is defined in Equation 2.


PS=P(X) (2)

Equation 2 shows that the propensity score for each household is the probability that each household receives the treatment (Z = 1), given the observed covariates (X). Observable variables also called covariates explain the characteristics of both benefitting households and non-benefitting. These covariates are broadly grouped into individual household characteristics, e.g., age and wealth characteristics, e.g., floor type.

These covariates are: $X_{1}$= Household size; $X_{2}$= Gender (1 = male; 0 = female); $X_{3}$= Marital Status (1 = married, 2 = cohabitating (not married), 3 = Divorced, 4 = Living apart not divorced, 5 = widow or widower, 6 = Not married); $X_{4}$= Current level of education (1 = No schooling; 2 = some primary; 3 = completed primary; 4 = some secondary;5 = completed secondary;6 = vocational; 7 = some university & 8 = completed university); $X_{5}$= Utilities (Total amount spent on electricity, water, garbage disposal, telephone, and landline bill); $X_{6}$= Type of Toilet (1 = Flush latrine/toilet with water; 2 = Traditional pit latrine (no water); 3=(Partly) open pit (no roof or no well); 4 = Communal latrine; 5 = None/bush (go into forest)); $X_{7}$ = Floor Material (1 = Mud/earth, 2 = Wood/plank, 3 = Tiles, 4 = Concrete/Cement, 5 = Grass, 5 = Other), $X_{8}$ = Roof material (1 = Mud, 2 = Thatch, 3 = Wood, 4 = Iron sheets, 5 = Concrete/Cement, 6 = Roofing tiles, 7 = Asbestos); $X_{9}$ = Wall material (1 = Mud/Mud brick, 2 = Stone, 3 = Burnt bricks, 4 = Concrete/Cement, 5 = Wood/Bamboo, 6 = Iron sheets, 7 = Cardboard,); $X_{10}$ = Energy source (1 = Kerosine/paraffin, 2 = Gas, 3 = Main electricity, 4 = Solar panels/private generator, 5 = Battery, 6 = Candles,7 = Firewood). *Treatment variable* used for Equation 2 is $X_{11}$=Benefitting Household (1 = Household is benefitting, 0 = Household is not benefitting) and the *outcome variable* is $X_{12}$ =Log of per-capita monthly expenditure on food items (Naira)

### Ethical approval

All procedures involving respondents adhered to the ethical standards of Akwa Ibom State University (AKSU) and ethical approval was granted by AKSU Research Ethics Committee. Informed consent was obtained orally from all the respondents before data collection.

## IV. Result

This section presents the result of the analysis and describes key findings. Household characteristics, food security status of beneficiaries and non-beneficiaries, impact derived from conditional cash transfer scheme and effect of conditional cash transfer on household food security are discussed in this section.

### Households characteristics

Household characteristics are present in Table 2. Over two-thirds of benefitting (65%) and non-benefitting (73%) households head are male while one-third of benefitting households (36%) and one-fourth of non-benefitting households are female-headed households. For household size, majority of the benefitting households (72%) had between 1–8 people while non-benefitting households had about 68% of people respectively.

**Table 2:** Socio-economic characteristics.

Households characteristics	Beneficiaries (%)	Non-beneficiaries (%)
**Gender**		
Male	64.44	73.33
Female	35.56	26.67
Total	100	100
**Household size**		
1-4	31.11	36.66
5-8	41.11	31.66
9-12	20.00	23.33
>12	7.77	8.33
Total	100	100
**Farm size**		
<1	18.88	30.00
1	7.78	10.00
2	31.11	25.00
>2	42.22	35.00
Total	100	100
**Educational level**		
No schooling	3.33	3.33
Some primaries	4.43	8.33
Completed primary	10.00	3.33
Some secondary	3.33	6.67
Completed secondary	38.89	26.67
Vocational	1.11	0
Some university	25.56	26.67
Completed university	14.44	23.33
Total	100	100
**Age**		
24-29	4.44 [26]	3.33 [21]
30-37	12.22 [34]	16.67 [34]
38-41	11.11 [40]	3.33 [40]
42-47	17.78 [45]	6.67 [46]
48-53	15.56 [51]	16.67 [49]
>53	38.89 [62]	53.33 [60]
Total [Total mean age]	100 [49]	100 [42]
**Energy source**		
Kerosene	42.22	47.46
Gas	4.44	3.39
Main electricity	23.33	20.34
Solar panels	2.22	3.39
Battery	16.67	11.86
Candles	10.00	13.56
Firewood	1.11	
Others		
Total	100	100
**Source of drinking water**		
Pipe water	26.67	35.00
Well/borehole protected	46.67	40.00
Well/borehole unprotected	1.77	–
Rivers, streams or dam	25.56	25.00
Total	100	100

One-third of benefitting households (38%) and non-benefiting households (35%) have small farms between 1–2 hectare. However, majority of both households (42% and 35% respectively) had farms larger than two hectares. Both households had a very low percentage of people who had no formal education (<4%) while majority (96%) had one form of formal education. Benefitting (39%) and non-benefitting (25%) households had completed secondary school, this was the largest education level compared to other categories. In terms of age, most household heads for both benefitting (39%) and non-benefitting households (53%) were older than 53 years with an average age of 62 and 60 years respectively. Kerosene was the major source of cooking energy used by both benefitting (42%) and non-benefitting households (48%). This was followed by electricity (23% and 20% respectively) and battery (17% and 12% respectively). Well or borehole (protected) was the main source of drinking water for both benefitting (47%) and non-benefitting households (40%). This was followed by pipe water (27% and 35%) and river (26% and 25%) for benefitting and non-benefitting households respectively.

### Food security status

**Fig 1:**
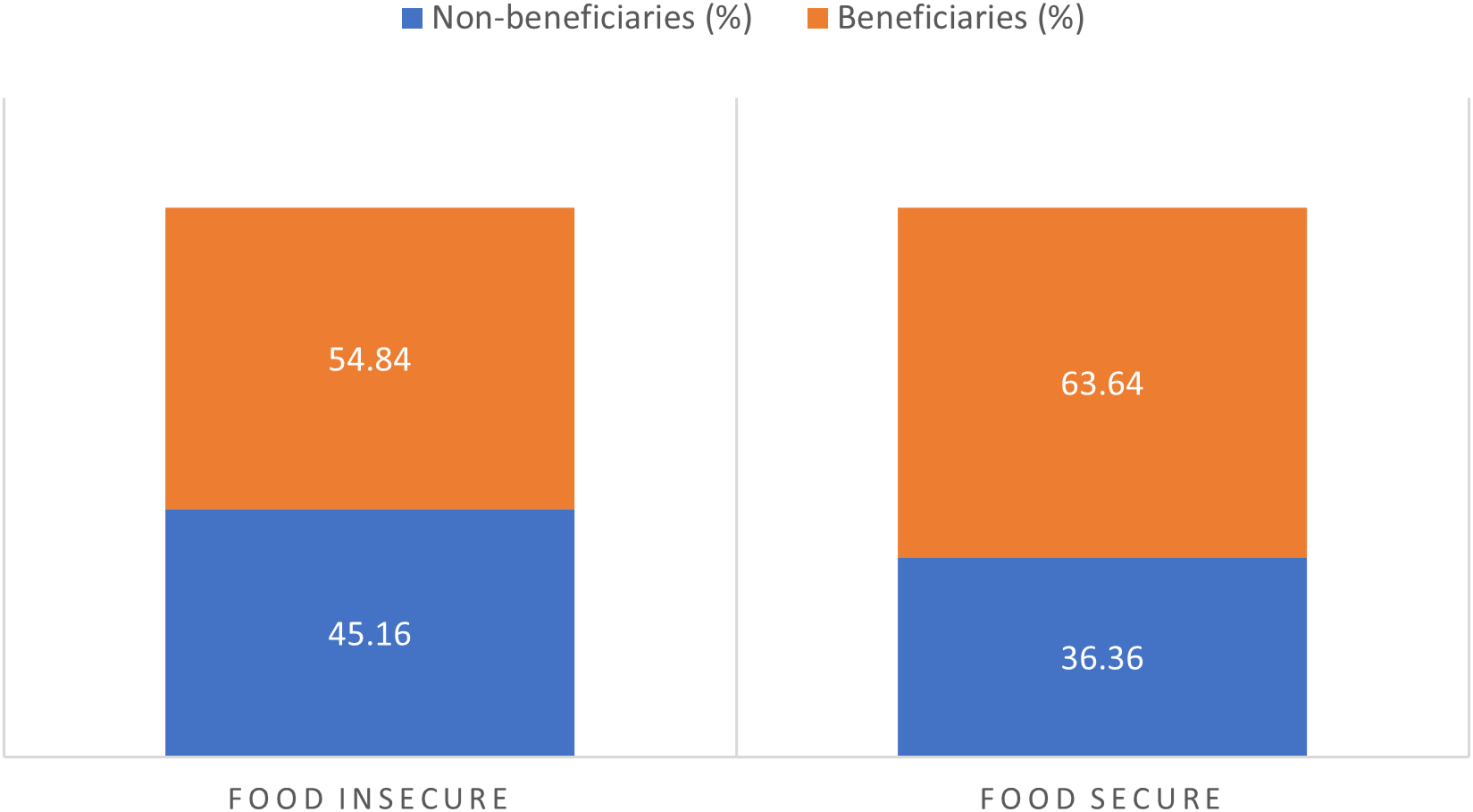
Comparing food security status.

**Fig 2:**
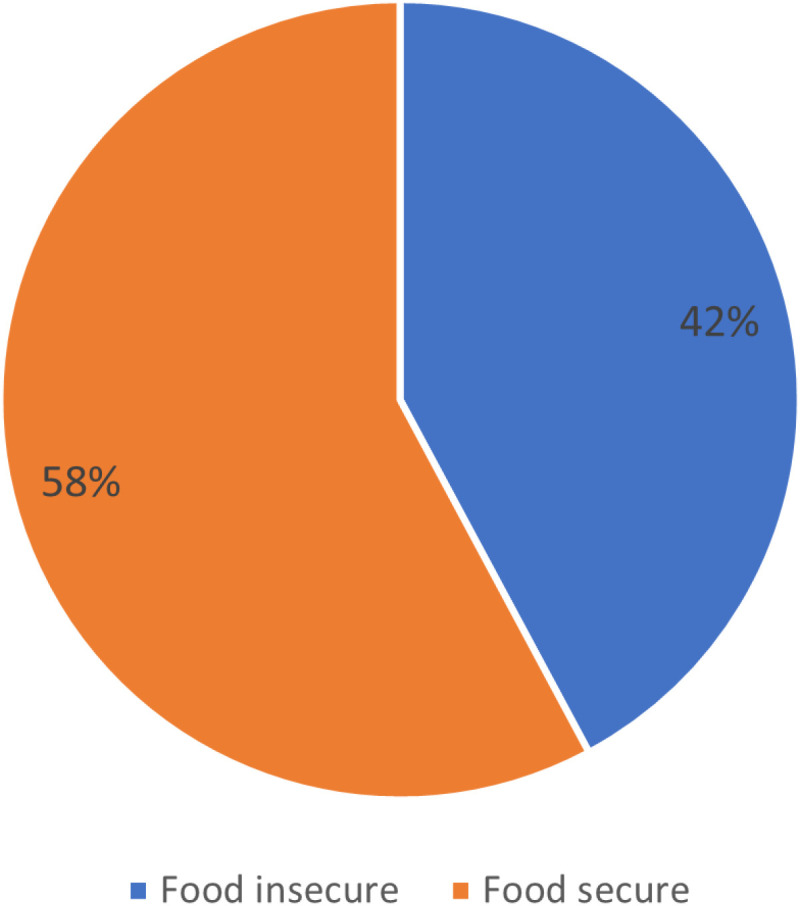
Overall food security status.

This section comparatively discuss the result of food security status of both the beneficiaries and non-beneficiaries of conditional cash transfer scheme of Household Upliftment Programme (HUP). The results are presented in Table 3, Fig 1 and Fig 2. As shown in Table 3, the overall food security line, which is 2/3 Mean Per Capita Expenditure (MPCE) for all the households is N4,203.23($10.06) while it is N4,224.12 ($10.12) and N4224.42 ($10.1.2) for benefitting and non-benefitting households respectively. The food security line for each category shows that there is little or no difference among MPCE for non-benefitting, benefitting or overall households. The food security threshold was used to determine the food security status of households as discussed in Section III. Table 3 also presents the descriptive statistics for the total amount spent on food and per capita food monthly expenditure. The result shows that on average, the overall amount spent on food per month is N33,110.13($79.31) while N6,304.846 ($15.19) is the amount spent on food per individual in a household per month. For benefitting and non-benefitting households, on average they spent about N31,917.78 ($76.46) and N34,898.67 ($83.60) respectively on food per month. While per capita food expenditure for benefitting and non-benefitting households per month were N6,304.66 ($15.19) and N6,305 ($15.10) respectively.

**Table 3:** Descriptive statistics of monthly food expenditure and per-capita food expenditure.

Food expenditure	Mean	Standard deviation	Minimum	Maximum
Total food expenditure (N)	33,110.13	16,077.87	3,500	10,220
Total food expenditure for beneficiaries (N)	31,917.78	16,122.09	3,500	95,300
Total food expenditure for non-beneficiaries (N)	34,898.67	15,978.41	9,800	102,200
Per-capita food expenditure	6,304.846	4,523.117	673.3333	27,266.67
Per-capita food expenditure for beneficiaries	6,304.66	4,202.95	673.33	20,600
Per-capita food expenditure for non-beneficiaries	6,305.11	5,001.96	895	17,266.67

Note: 2/3 MPCE for all households is N4203.23; 2/3 MPCE for a benefitting and non-benefitting household are N4224.12 and N4224.42 respectively. Exchange rate August 2022: $1 = N417.47 (source:https://www.oanda.com/currency-converter/en/?from=USD&to=NGN)

Fig 1 compares the food security status of both beneficiaries and non-beneficiaries households. Comparatively, the result shows that food insecurity is higher for benefitting households (55%) than non-benefitting households (45%). In contrast, benefitting households had a higher proportion of food-secure households (64%) than non-benefitting households (36%). Overall, 58% of households in the study population were food secure while 42% were food insecure (Fig 2).

### Impact of conditional cash transfer of HUP on beneficiaries

The impact derived from Conditional Cash Transfer scheme of HUP in the study area is represented in Table 4. The threshold mean is 2.0, meaning that if the derived benefit has a mean of 2.0 and above then such benefit has a high impact on the beneficiaries. In this table, all the benefits derived from the cash transfer programme are all significant but only vary by the ranking of the impact. Training and empowerment are ranked first and have the highest impact, this was followed by business boost, new business establishment, increase self-confidence, lower level of dependency, better inclusion in groups, and increase psychosocial support.

**Table 4:** Impact derive from conditional cash transfer scheme of Household Upliftment Programme.

Benefit Derived	Highly Beneficial	Beneficial	Not Beneficial	Total	Weighted mean	Std dev	Rank
Training and empowerment	82	6	2	90	3.02	2.42	1^st^
New business establishment	57	30	3	90	2.60	2.11	3^rd^
Business boost	66	21	3	90	2.70	2.20	2^nd^
Lower level of dependency	49	39	2	90	2.52	2.03	4^th^
Increase psychosocial support	36	50	4	90	2.36	1.87	6^th^
Better inclusion in groups	43	43	4	90	2.43	1.95	5^th^
Increased self-confidence	59	26	5	90	2.60	2.12	3^rd^

### Effect of the conditional cash transfer on household food security

The result of the effect of conditional cash transfer on households’ food security of the beneficiaries compared to household food security of the non-beneficiaries using propensity score analysis is presented in Table 5 and Table 6.

**Table 5:** Propensity Score post-estimation result.

Variables	Unmatched	Mean		%bias	%Reduct bias	t-test	
	Matched	Treated	Control			T	P > |t|
**Household Size**	U	7.087	7.509	−10.1	−54.5.5	−0.55	0.581
	M	7.087	6.434	15.6		1.07	0.288
**Age**	U	49.435	50.418	−8.2	−62.1	−0.45	0.653
	M	49.435	51.029	−13.2		−0.77	0.445
**Gender**	U	1.289	1.290	−0.2	−6666.0	−0.01	0.990
	M	1.289	1.217	15.8		0.97	0.332
**Marital Status**							
Widow or widower	U	0.159	0.272	−27.6	87.2	−1.54	0.126
	M	0.159	0.173	−3.5		−0.23	0.821
Not married	U	0.579	0.0363	10.1	−34.1	0.55	0.582
	M	0.570	0.0289	13.6		0.83	0.407
**Education**	U	5.536	5.654	−6.1	−145.0	−0.34	0.734
	M	5.536	5.826	−14.9		−0.79	0.433
**Utilities**	U	1240.6	983.64	36.8	70.1	1.99	0.049^**^
	M	1240.6	1163.8	11.0		0.57	0.572
**Toilet**	U	2.898	2.290	43.8	11.8	2.41	0.018^**^
	M	2.898	2.362	38.7		2.42	0.017^**^
**Floor material**							
Thatch	U	0.130	0.036	34.3	38.4	1.84	0.068^*^
	M	0.130	0.072	21.1		1.12	0.263
Wood	U	0.028	0.127	−36.9	70.5	−2.12	0.036^**^
	M	0.028	0	10.9		1.42	0.157
Iron Sheet	U	0.463	0.6	−27.3	−166.0	−1.51	0.133
	M	0.463	0.826	−72.7		−4.77	0.000^***^
**Roof material**	U	3.463	4.181	−87.0	85.9	−4.69	0.000^***^
	M	3.463	3.362	12.3		0.60	0.548
**Wall material**	U	4.231	4.345	−15.2	−40.4	−0.81	0.417
	M	4.231	4.391	−21.3		−1.25	0.215
**Energy source**	U	2.463	2.709	−13.3	29.1	−0.74	0.461
	M	2.463	2.289	9.4		0.55	0.583
**Sample**	**Ps R2**	**LR chi2**	**p > chi2**	**MeanBias**	**MedBias**	**B**	**R**
Unmatched	0.300	51.02	0.000	24.7	19.1	140.5^*^	2.00^*^
Matched	0.063	6.78	0.964	12.1	10.9	59.6^*^	1.50^*^

* significant at 10%; ^**^significant at 5% and ^***^significant at 1%

**Table 6:** Treatment effect result of different propensity matching methods.

Treatment Effect Estimators	ATET	Standard Error	Bias After Matching	Off Support
NN with replacement	−0.2307	0.2539	20.8	0
NN without replacement	−0.0142	0.1420	18.1	14
NN with replacement using logit regression	−0.2443	0.2426	21.9	0
NN with caliper 0.1	−0.2610^*^	0.2017	14.9	30
Radius with caliper 0.01	−0.2137	0.2235	11.8	30
Kernel with bandwidth 0.06	−0.0850	0.2101	9.3	14
Kernel with bandwidth 0.01	−0.2261	0.2246	12.5	30

* significant at 10%

As presented in Table 5, propensity score analysis matches households based on the probability score. Table 5 shows how households in the treatment group are therefore compared with those in the control group who have similar propensity scores and the average difference across the matched individuals (shown in Table 6). Furthermore, the result assesses the quality of matching between households who were selected to receive the N5000 ($11.98) and those who were not selected. The table presents useful results to investigate the covariates that are not very comparable between the two groups.

The result in Table 5 shows that before and after matching the following covariates were not significant: household size, age, gender, marital status, education, wall material and energy source. This means that these covariates which are mostly household characteristics are not comparable between the two groups given the outcome variable. Whereas utilities, type of toilet, floor material and roof material were significantly at different levels. This implies that these covariates are the ones that are comparable between the treatment group and the control group.

Nevertheless, Table 5 also showed a set of statistics called the bias (before and after matching). The bias refers to the difference in the characteristics of the two groups and not bias in the actual treatment effect estimates [35]. The result showed that for each variable the bias is greatly reduced and that there is no significant difference after matching. The bias reduces on average from 24.7% to 12.1%. The bias value of 12.1% falls outside the 3–5% bias range which is considered to be the acceptable region [36]. Also, the table shows a reduction in the pseudo-r-square of the probit model of the likelihood of being in the treatment group before (using the whole sample) and after matching (using only those in the matching process) and for the matched sampled. The pseudo-r-square reduced from 30.0% (significant at 1%) to 6.3% (not significant) which is what is expected given that after matching not all households were able to be paired.

Table 6 shows the treatment effect result using different propensity score matching methods. Simply, the result shows the effect of conditional cash transfer on food security status of the respondent. The propensity score result is used to evaluate the result of the conditional cash transfer programme of household upliftment programme on food security. The result is used to evaluate the impact of a “treatment” (those households that received the conditional cash transfer) on an outcome variable (which is monthly food expenditure that is used to determine the food security status of the respondents). The ATET is a common measure that is to evaluate the impact of a treatment. The Average Treatment Effect on the Treated (ATET) is the expected treatment effect for those in the treatment group [33]. It only examines the difference in outcomes for those in the treatment group. The result showed that all the different treatment effect estimators were not significant except the nearest neighbour matching with calliper 0.1 which is significant at 10%. Nearest neighbour matching selects for matching households in the control group to households in the treatment group whose propensity score is close to that of households in the treatment group [33,36]. With a caliper of 0.1, the nearest neighbour’s matching is restricted to a tolerated distance of 0.01, i.e., difference in the propensity score must fall within this distance in which a match can be found. If there is no match for a household in the treatment group based on a distance of 0.01 then the observation is discarded [33,36].

The result of the nearest neighbour matching with calliper 0.1 indicates that 30 households were off support. In other words, 30 households were not matched because they fell outside the calliper distance of 0.1. The biase after matching is 14.9% which is also higher than the acceptable range of 2–3%. However, the ATET estimator is −0.2610 and this measures on average the difference between the treatment effect before and after matching. This means that average treatment effect of the conditional cash transfer on household food security of the treatment group compared to the control group is −26.10%. It means that on average the difference between food security status of benefitting households compared to non-benefitting households is −26.10%. So, on average households that did not benefit from the condition cash transfer scheme spend 26.10% more on food than households that benefitted from the conditional cash transfer scheme.

## V. Discussion

On food consumption, the findings showed that households spend a significant amount of money on food per month. On average, benefitting and non-benefitting households spent about N31,917.78 ($76.46) and N34,898.67 ($83.60) respectively on food per month. This might suggest that households that benefit from conditional cash transfers programme may have slightly lower food expenditures on average. This finding implies that while the programme may be effective in reducing poverty and improving food security among the poor, the impact may vary depending on the design and implementation of the programme. Furthermore, the fact that benefitting households have lower food expenditures on average may not necessarily indicate that they are worse off, but rather that they can prioritize other basic needs such as healthcare and education [37–40].

The findings that food insecurity is higher among benefiting households compared to non-benefiting households in the state raise critical questions regarding the effectiveness of conditional cash transfer (CCT) programs. One plausible explanation for this unexpected outcome is the inadequacy of the cash transfers provided to these households. Research has shown that insufficient cash transfers can fail to meet the dietary needs of beneficiaries, leading to persistent food insecurity despite the financial assistance received [41,42]. Furthermore, it has been suggested that CCT programs may not adequately target the most vulnerable households, which could exacerbate food insecurity among those who are supposed to benefit from such interventions [43]. Conversely, the observation that benefitting households exhibit a higher proportion of food-secure households than their non-benefiting counterparts suggests a potential positive impact of CCT programs on food security. This aligns with findings from various studies that indicate cash transfers can enhance food access and dietary diversity among beneficiaries [1,41]. For instance, Eluwa’s study highlights that cash transfers significantly improved household dietary diversity and reduced hunger scores, despite some households experiencing a decline in living standards [41]. This suggests that while CCTs may not completely eliminate food insecurity, they can contribute positively to the food security landscape when designed and implemented effectively.

However, it is worth noting that the proportion of food-insecure households among beneficiaries in this study is still quite high, indicating that there is substantial room for improvement in the design and execution of these programs. The literature indicates that merely providing cash transfers is not sufficient; the structure of these programs must also address underlying issues such as poverty, unemployment, and local food prices, which can undermine food security efforts [1,42]. For example, studies have shown that cash transfers can inadvertently increase local food prices, making it more difficult for non-beneficiaries to access food, thereby worsening food insecurity in the broader community [1,42]. The alarming statistic that 42% of households in the study area were food insecure underscores the urgent need for enhanced interventions. Expanding food assistance programs, specifically targeting the most vulnerable households, and addressing systemic issues such as poverty and unemployment are critical steps that could significantly improve food security outcomes in Akwa Ibom State [1,41]. The evidence from various contexts suggests that a multifaceted approach, combining cash transfers with food assistance and other social protection measures, is likely to yield better results in combating food insecurity [44].

The result of this study also showed that the CCT facilitated the beneficiary to invest in human and other productive assets which can provide a way out of persistent and intergenerational food security. For instance, training and empowerment had the highest impact. This is mainly because beneficiaries’ participation and training were done in groups. Through the programme training beneficiaries are exposed to information and education on life skills and financial literacy as well as micro business they can start up in their communities. Empowerment and training have been proven to be effective ways of improving the livelihoods of beneficiaries of development programmes [45]. This is because training and empowerment allow beneficiaries to take control of their own lives and become self-sufficient. By providing training on life skills and financial literacy, beneficiaries are better equipped to manage their finances and start small businesses in their communities. The HuP permits beneficiaries to form economic groups and cooperative societies, which is one of the core benefits of the cash transfer programme. These groups provide opportunities for beneficiaries to pool their resources, share knowledge, and collectively engage in income-generating activities. The success of these groups has been demonstrated in various studies, which have shown that they lead to increased economic opportunities, productivity, access to credit, and social inclusion among beneficiaries [8,46,47]. HuP has brought financial inclusion to many of its beneficiaries, who were previously unaware of banking services due to their limited financial resources. Many of these beneficiaries were struggling to meet their basic needs, let alone think about saving. However, through the programme, they have been introduced to livelihood activities that help them generate more income, encouraging them to develop a habit of saving. As a result, many of them have now opened bank accounts to save their earnings, providing them with greater financial stability and security for the future. Psychosocial support occurs through peer review and cohesion of beneficiaries. Through the groups, beneficiaries build positive synergy among themselves. They learn from each other as well as monitor each other, resolve conflict and facilitate decision-making. Psychosocial interventions are very effective in eliminating extreme poverty. Because it builds the skills of the beneficiaries and strengthens the instrumental and normative support they receive from their households and community [47]. Furthermore, through involvement in the programme, some beneficiaries who could not send their children to school can now do so. They can afford their children’s exercise books, sandals and other school accessories. Similar studies have found that cash transfer improves the educational outcomes of children in Tanzania and Nigeria by improving children’s school participation and performance [11,41].

Regarding the impact of conditional cash transfer on protecting the beneficiary’s basic level of food consumption from becoming food insecure. Our result showed that the Average Treatment Effect on the Treated (ATET) estimator value of −0.2610 reflects the average difference in outcome (food security) between the treated and control groups after matching. A negative value suggests that, on average, the conditional cash transfer had a detrimental effect on the food consumption of the beneficiaries compared to non-beneficiaries. On average, benefiting households spend 26.10% less on food consumption than non-benefitting households. This finding differs from other studies which showed that conditional cash transfer programmes increase the quantity and quality of food consumed [17,38,41]. However, a similar finding to this research was reported by [48], who evaluated the impact of Livelihood Empowerment Against Poverty Programme (LEAP) – a social cash transfer program that provides cash and health insurance to extremely poor households in Ghana. The result showed that LEAP had a zero impact on household food consumption. In terms of food security, this result further means that benefiting households are worse off compared to non-benefitting households. The reason for this is that N5000 ($11.98) paid monthly to households are not paid regularly. As at the time this research was carried out, the government were still owing benefitting households eight months stipends. Also, the N5,000 ($11.98) is not commensurate with the skyrocketing inflation in goods and services experienced in the country. This result does not mean that the conditional cash transfer does not have any positive effect on the beneficiary but that with respect to food security benefitting households are worse off than non-benefitting households. There is a need for the government to further appraise this programme and adjust the cash to accommodate the current inflation in the price of goods and services. The implication of the finding regarding the detrimental effect of conditional cash transfers on food consumption raises important questions about the effectiveness of such programs in addressing food insecurity. It also highlights the need for careful consideration of the design and implementation of cash transfer programs to ensure their positive impact on beneficiaries’ well-being.

This paper is subject to certain limitations. The study’s total sample size was limited due to insufficient funding, which weakened the research. Consequently, the findings of this study may not be generalizable to the entire population. A larger sample size would have yielded more reliable and robust results, facilitating a more in-depth analysis and greater generalizability of the findings. Nonetheless, to ensure equal representation, the research employed a simple random sampling technique. Future research should be conducted with a representative sample, necessitating adequate funding for the research.

## VI. Conclusion

The study investigated the impact of Condition Cash Transfer of Households Uplifting Programme on household food security in Akwa Ibom State, Nigeria. The study specifically investigated the impact of CCT on protecting the beneficiary’s basic level of food consumption from becoming food insecure and also determined if CCT of HUP facilitate the beneficiary to invest in human and other productive assets which can provide a way out of persistent and intergenerational food security.

The study revealed several key findings. Benefitting households spend slightly less on food compared to non-benefitting households, indicating that CCTs allow these households to prioritize other basic needs such as healthcare and education. However, despite the cash transfer, food insecurity remains higher among benefitting households indicating that the cash transfers provided may be inadequate to meet dietary needs, and the targeting of the most vulnerable households may be insufficient. Furthermore, the irregularity and insufficiency of the cash transfers, coupled with high inflation, have limited the program’s effectiveness in improving food security. Benefitting households spend 26.10% less on food consumption than non-benefitting households, highlighting the need for adjustments in the program’s design and implementation.

The study also reveals that the CCT program has facilitated beneficiaries to invest in human and productive assets. The training and empowerment provided have improved livelihoods; the formation of economic groups and cooperative societies have enhanced financial and social inclusion; the introduction of financial literacy and saving habits has made financial services accessible to many and the psychosocial support has fostered a sense of community among beneficiaries.

In conclusion, while the conditional cash transfer program of HuP has shown the potential to positively impact beneficiaries, the high levels of food insecurity among beneficiaries in the state highlight the need for a re-evaluation of the program. Future efforts should focus on ensuring that cash transfers are adequate, effectively targeting the most vulnerable populations, and addressing broader socio-economic factors that contribute to food insecurity.

### Implications for policy and practice

Concerning the implication of this study’s findings for policy and practice, it is essential to prioritize a few key areas. First and foremost, there is a need to improve the targeting mechanisms of the CCT to ensure that the most vulnerable households receive the necessary support from the program. Furthermore, it is imperative to ensure adequate funding by increasing the amount of cash transfers to account for inflation and to guarantee regular disbursements. Moreover, taking a comprehensive approach is essential. This means combining cash transfers with other social protection measures, such as food assistance and employment programs, to tackle underlying issues like poverty and unemployment. By addressing these areas, the CCT program can make a more meaningful impact on food security and help create a stronger, more resilient community.

### Future research

Future research needs to focus on the following areas. Firstly, a long-term (longitudinal) study is needed to assess the sustained impact of CCT programs on food security and overall well-being. Secondly, there is a need for comparative analysis to assess the effectiveness of different CCT program designs and implementation strategies in various regions. Lastly, a holistic evaluation should be conducted to explore the combined effects of cash transfers and other social protection measures on food security and poverty alleviation.
